# Immune Checkpoint Inhibitor–Induced Effusive–Constrictive Pericarditis: The Role of Multimodality Imaging

**DOI:** 10.1016/j.cjco.2026.04.004

**Published:** 2026-04-22

**Authors:** Magdaline Zawadka, Davinder Jassal, Ahmad Didi, Iain Kirkpatrick, Robin Ducas

**Affiliations:** Department of Cardiology, University of Manitoba, Winnipeg, Manitoba, Canada

**Keywords:** effusive constrictive pericarditis, immune checkpoint inhibitor, multimodality imaging

**Immune checkpoint inhibitors (ICIs) have transformed cancer therapy, offering robust clinical benefits across a wide range of malignancies.****^1^** **As their use expands into earlier disease stages and combination regimens, recognition of potential immune-related adverse events has become increasingly important. Although infrequent, cardiovascular toxicities from ICIs can be clinically significant, encompassing myocarditis, pericarditis, heart failure, and arrhythmias. Pericardial involvement represents a rare manifestation with diverse clinical and imaging features.****^2^**

**We present a rare case of ICI-associated effusive–constrictive pericarditis (ECP) characterized by distinctive echocardiographic and cardiac magnetic resonance (CMR) findings, highlighting the pivotal role of multimodality imaging in diagnosis and management.**

## Case Report

A 66-year-old female patient with renal cell carcinoma presented with hypoxia and hypotension following an elective gastroscopy performed at a peripheral hospital, for evaluation of gastrointestinal symptoms. She was recently started on pembrolizumab and had completed 3 cycles. Initial computed tomography (CT) of the chest identified a large unilateral pleural effusion, for which a pigtail catheter was inserted in the emergency department. Clear fluid—1.2 L—was aspirated and sent for analysis, including cultures and cytology. The CT also demonstrated a large pericardial effusion. Notably, a chest radiograph obtained 2 weeks earlier revealed a normal cardiac silhouette with small bilateral pleural effusions. A CT of the chest performed 4 months prior showed no evidence of pleural or pericardial effusions, and no metastatic disease, suggesting a subacute onset. Physical examination findings were concerning for cardiac tamponade, prompting transfer to our tertiary-care centre for urgent assessment.

**Figure 1:**
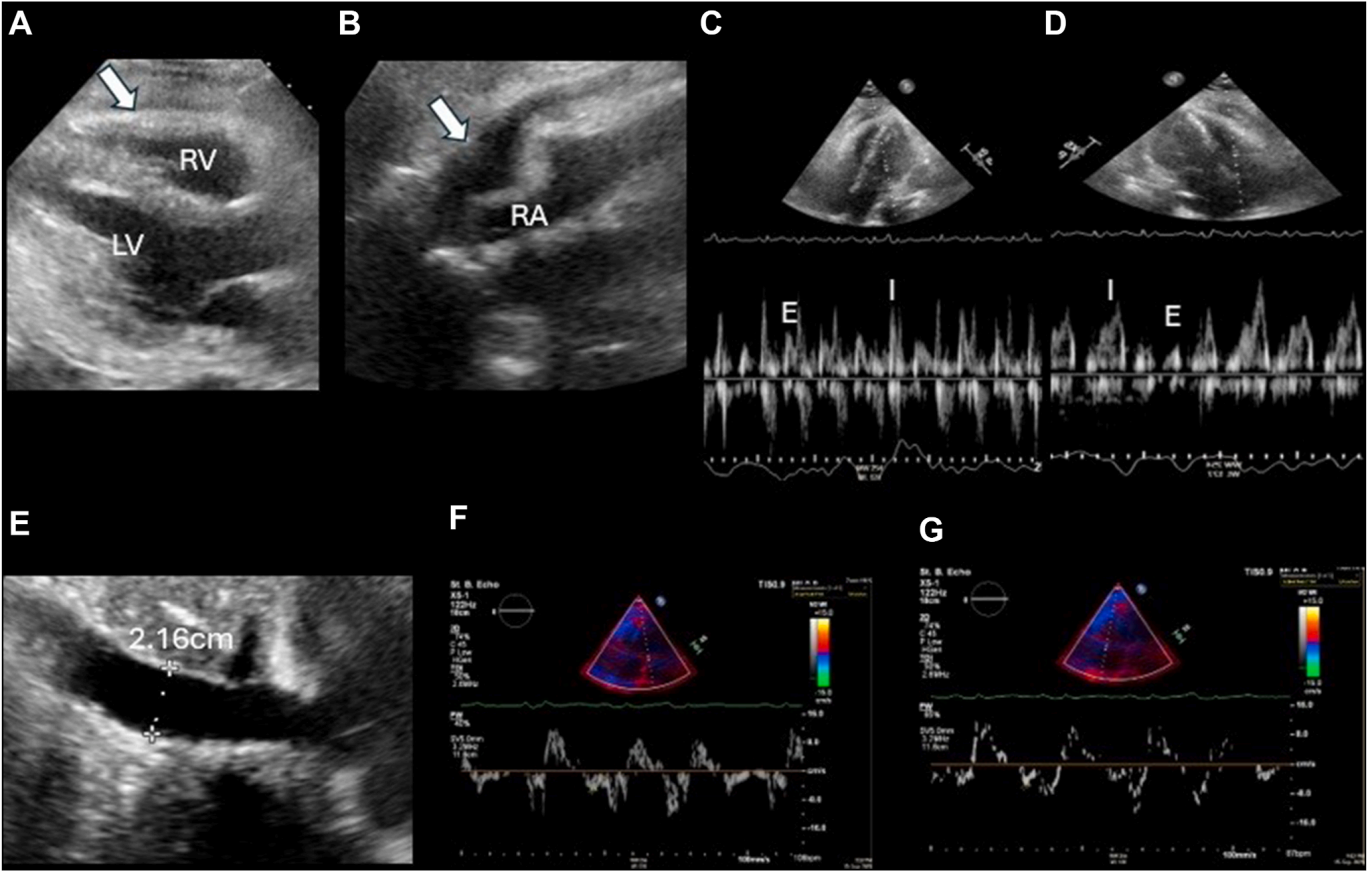
Echocardiographic findings of effusive-constrictive pericarditis. (**A**) Parasternal long-axis (PLAX) view revealing a thick pericardium alongside the right ventricle (RV; **arrow**). (**B**) Subcostal view with pericardial effusion causing right atrial (RA) diastolic collapse (**arrow**). (**C, D**) Mitral valve and tricuspid valve respirophasic inflow variation. (**E**) Dilated inferior vena cava measuring 2.16 cm. (**F, G**) Annulus reversus with the septal e’ velocity (6.4 cm/s) being higher than the lateral e’ (5.8 cm/s). E, expiration; I, inspiration; LV, left ventricle.

On arrival, she remained hypotensive (80/44 mm Hg) and hypoxic (oxygen saturation 95% on 4 L/min via nasal cannula). High-sensitivity troponin was mildly elevated at 39 ng/L, peaking at 72 ng/L the following day, and her inflammatory markers were markedly elevated (C-reactive protein 270 mg/L). Blood cultures were obtained, and empiric antibiotics were initiated for a possible infectious etiology. Transthoracic echocardiography revealed a small-to-moderate pericardial effusion with a thick echodense circumferential layer within the pericardial space. Respirophasic ventricular septal shift, a dilated inferior vena cava (IVC) with reduced respiratory variation, and annulus reversus were also observed, suggestive of ECP (Fig. 1). No prior cardiac imaging was available for comparison.

Given her hemodynamic instability and echocardiographic findings, she was admitted to the coronary care unit and treated for obstructive shock secondary to ECP. Viral serologies and an autoimmune workup were sent to evaluate for alternative etiologies of her pericardial disease. Right heart catheterization was attempted, but measurements were limited by the patient’s inability to perform breath-holding maneuvers and worsening hypoxia during the procedure. Despite these limitations, her cardiac index was preserved at 2.8 L/min/m^2^, with elevated right atrial and pulmonary capillary wedge pressures of 17 mm Hg and 26 mm Hg, respectively.

Due to the complexity of her presentation, respirology was consulted for further evaluation of the pleural effusion. Pleural fluid analysis demonstrated a lymphocyte-predominant effusion, but the rapid onset made a malignant etiology less likely, and findings were not consistent with bacterial infection. Overall, the presentation was felt to be most consistent with ICI-mediated serositis. As no clear evidence of cardiac tamponade was present, pericardiocentesis was deferred. Cardiac surgery was consulted, and they felt that a conservative management approach was best.

Following multidisciplinary discussion with oncology, ICI-related pericardial disease was deemed the most likely etiology. High-dose corticosteroid therapy was initiated following oncology’s recommendations with intravenous methylprednisolone 1 g daily for 3 days, followed by oral prednisone at 1 mg/kg with a gradual taper of 10 mg per week. Colchicine was initially deferred due to her gastrointestinal symptoms and an oliguric acute kidney injury.

**Figure 2:**
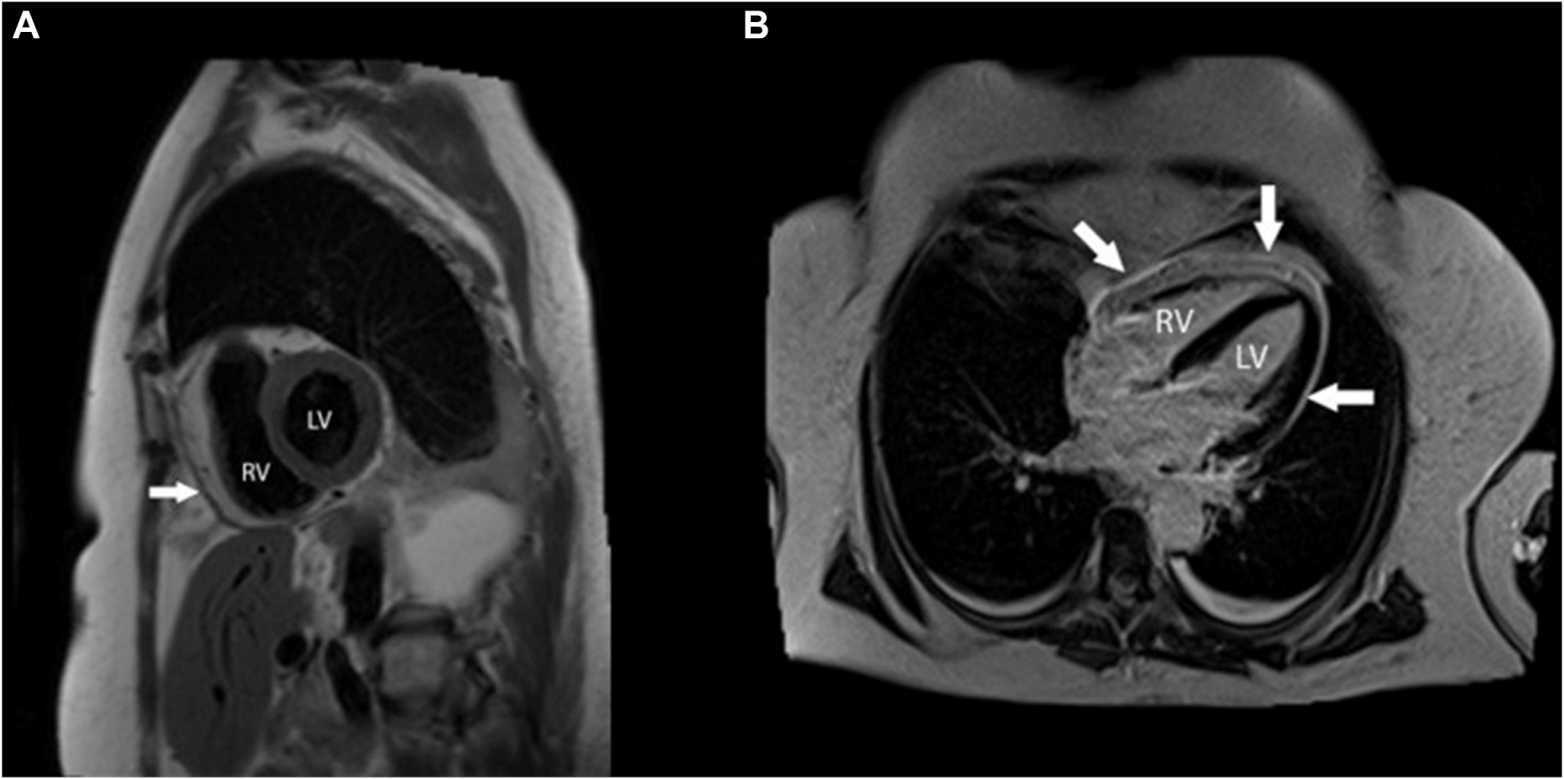
Cardiac magnetic resonance findings. (**A**) Short-axis T1-weighted turbo spin-echo image shows a thickened (4 mm) pericardium overlying the right ventricle (RV; **arrow**). (**B**) Four-chamber T1-weighted gradient echo late gadolinium enhancement image demonstrates circumferential enhancement of the pericardium (**arrows**). LV, left ventricle.

CMR was performed once her kidney function returned to normal. The imaging demonstrated a mild septal bounce and a trivial pericardial effusion. Thickening and hyperenhancement of the pericardium was present, consistent with acute pericarditis (Fig. 2). No late gadolinium enhancement of the myocardium occurred, excluding concomitant myocarditis. Overall, the findings were consistent with ECP in the setting of acute pericarditis.

With treatment, her C-reactive protein level decreased to 17 mg/L with therapy, and blood and pleural fluid cultures remained negative. Cytology from the pleural fluid was also negative. Her autoimmune panel was unremarkable, including negative serologic markers for lupus and rheumatoid arthritis, with normal complement and immunoglobulin levels. Viral serologies were also negative for hepatitis B virus, hepatitis C virus, , HIV, Epstein-Barr virus, cytomegalovirus, and parvovirus. She never developed any arrhythmic complications and improved clinically, allowing for discharge home to complete her steroid taper.

At a 3-month follow-up evaluation in the cardio-oncology clinic, she reported recurrence of chest pain while tapering prednisone from 30 mg to 25 mg daily, prompting an increase back to 40 mg daily. She also endorsed persistent exertional dyspnea. Point-of-care ultrasound revealed residual organized pericardial material, but the inferior vena cava was normal in size with preserved respiratory variation and no features of constriction. In keeping with European Society of Cardiology guideline recommendations, a slower steroid taper was initiated over 6-8 weeks (reduction to 30 mg, followed by decrements of 1 mg every 3 days to 20 mg, then 1 mg weekly). Colchicine 0.6 mg twice daily was introduced to facilitate steroid weaning.

Repeat CMR showed resolution of the septal bounce but persistent pericardial hyperenhancement, thought to represent fibrosis rather than active inflammation. At a subsequent follow-up evaluation 3 months later, she remained on prednisone 11 mg daily and colchicine without recurrence of her chest pain. She will continue her steroid taper and be reassessed in 1 year, with planned discharge from the clinic if she remains clinically stable. Further chemotherapy is not planned, given the absence of residual malignancy, and rechallenging with ICI therapy is contraindicated in light of her presentation.

## Discussion

In patients with malignancy, pericardial effusions most commonly result from malignant infiltration, although they also may occur secondary to treatment with traditional chemotherapy, radiation therapy, or targeted therapies.^3^ Immunotherapy has recently emerged as a novel cancer treatment, and it has been implicated increasingly in pericardial immune-related adverse events. However, data on ICI-related pericarditis or pericardial effusion remain limited, and to our knowledge, ECP related to immunotherapy has not been described previously.

Although pericardial disease may occur with any ICI, programmed death protein 1 (PD-1) inhibitors such as pembrolizumab and nivolumab demonstrate the strongest association with pericardial involvement. This association may reflect the key role of the PD-1 pathway in maintaining peripheral immune tolerance, such that its inhibition results in enhanced tissue-level T-cell activation and an increased propensity for organ-specific immune-mediated inflammation.^3^

ICI-associated pericardial disease can present acutely with variable symptoms, warranting a high index of suspicion. Prompt recognition and differentiation from malignant, infectious, and uremic causes is essential, as management differs substantially. In contrast to the chronic progression of malignant effusions, ICI-induced ECP typically reflects an acute, reversible inflammatory process, as seen in this case.

Our case highlights the diagnostic importance of multimodality imaging. CMR demonstrated pericardial inflammation and thickening, quantified inflammatory burden, and excluded concomitant myocarditis. Severe pericardial inflammation was confirmed by the late gadolinium enhancement severity criteria, defined as ≥ 50% circumferential extent across ≥ 3 slices, and > 3 mm thickness.^4^ The profound pericardial thickening observed in our patient emphasizes the acute inflammatory potential of ICI therapy and the reversibility of ECP with timely immunosuppression.

Notably, our patient developed ECP manifesting as obstructive shock, which is classified as a grade-4 immune-related adverse event (irAE). Grade-4 immune-mediated toxicities are defined as life-threatening events requiring urgent intervention and generally warrant permanent discontinuation of ICI therapy.^5^ Although irAEs are common with immunotherapy, grade-4 toxicities are rare, occurring in less than 1% of patients. Severe cardiovascular irAEs are particularly rare, but they are associated with significant morbidity and mortality and warrant prompt recognition and management.^2^

**Novel Teaching Points**

• ICIs can cause pericardial disease in the absence of concurrent myocarditis. Although ICI-related myocarditis is well described, this case demonstrates that isolated pericardial inflammation can result in constrictive physiology, highlighting the importance of considering ECP in patients presenting with acute hemodynamic compromise on ICIs.
• Multimodality imaging is essential for the diagnosis and management of ECP. Transthoracic echocardiography can identify respirophasic ventricular septal shift, annulus reversus, and inferior vena cava dilatation, whereas CMR allows detailed assessment of pericardial thickness, quantifies inflammation, and excludes myocardial involvement, guiding targeted immunosuppressive therapy.
• ICI-related ECP can present acutely as obstructive shock and represents a grade-4 irAE. Early recognition is crucial, as prompt initiation of high-dose corticosteroids can reverse pericardial inflammation and prevent disease progression. In general, grade-4 toxicities, such as obstructive shock, warrant permanent discontinuation of ICIs.

## Ethics Statement

The research reported in this paper adhered to the Declaration of Helsinki.

## Patient Consent

The authors confirm that a patient consent form has been obtained for this article.

## Funding Sources

The work did not receive any specific grant from funding agencies in the public, commercial, or not-for-profit sectors.

## Disclosures

The authors have no conflicts of interest to disclose.
